# Potentiodynamic Electrochemical Impedance Spectroscopy of Polyaniline-Modified Pencil Graphite Electrodes for Selective Detection of Biochemical Trace Elements

**DOI:** 10.3390/polym14010031

**Published:** 2021-12-22

**Authors:** Adel Yavarinasab, Mostafa Abedini, Hamed Tahmooressi, Sajjad Janfaza, Nishat Tasnim, Mina Hoorfar

**Affiliations:** 1 School of Engineering, University of British Columbia, Kelowna, BC V1V 1V7, Canada; Adel.yavarinasab@ubc.ca (A.Y.); htahmooressi@gmail.com (H.T.); sajad.janfaza@gmail.com (S.J.); 2 Abidi Pharmaceuticals, Research and Development Centre, Tehran 1389776363, Iran; m.abedini.eng@gmail.com; 3 Faculty of Engineering and Computer Science, University of Victoria, Victoria, BC V8W 2Y2, Canada; nishattasnim@uvic.ca

**Keywords:** polyaniline, graphite, electrochemical sensors, conductive polymers, impedance, trace elements

## Abstract

In this study, we analyzed the application of potentiodynamic electrochemical impedance spectroscopy (PDEIS) for a selective in situ recognition of biological trace elements, i.e., Cr (III), Cu (II), and Fe (III). The electrochemical sensor was developed using the electropolymerization of aniline (Ani) on the surface of the homemade pencil graphite electrodes (PGE) using cyclic voltammetry (CV). The film was overoxidized to diminish the background current. A wide range of potential (V = −0.2 V to 1.0 V) was investigated to study the impedimetric and capacitive behaviour of the PAni/modified PGE. The impedance behaviors of the films were recorded at optimum potentials through electrochemical impedance spectroscopy (EIS) and scrutinized by means of an appropriate equivalent circuit at different voltages and at their corresponding oxidative potentials. The values of the equivalent circuit were used to identify features (charge transfer-resistant and double layer capacitance) that can selectivity distinguish different trace elements with the concentration of 10 μM. The PDEIS spectra represented the highest electron transfer for Cu (II) and Cr (III) in a broad potential range between +0.1 and +0.4 V while the potential V = +0.2 V showed the lowest charge transfer resistance for Fe (III). The results of this paper showed the capability of PDEIS as a complementary tool for conventional CV and EIS measurement for metallic ion sensing.

## 1. Introduction

The presence of certain metal ions is an unmet need to drive biological processes in the human body: Any imbalances in that regard can cause serious disorders and malfunctions for living tissues. Among the metal cations, Cu (II), Cr (III), and Fe (III) receive significant attention due to their role in different metabolisms including roles in enzyme catalysis, heme synthesis, electron and oxygen transport, DNA repair and synthesis (iron-sulfur (Fe–S) clusters), and free-radical detoxification [1,2]. Apart from the metabolic role of heavy metal ions, one other aspect is that they are usually produced by anthropogenic activities and categorized as pollutants in the environment [3]. What makes it worse is the non-biodegradability of metal ions, which makes them accumulate in living organisms and cause harmful disorders [4]. The important role of metal ions in the body and environment necessitates the development of highly sensitive and selective techniques for trace level detection of the ions in different biological and environmental samples.

Generally, spectral, optical, and electrochemical methods are major approaches used to determine the ions in aqueous solutions. Inductively coupled plasma, coupled with mass spectroscopy, optical, and/or atomic emission spectrometry [5] as well as X-ray fluorescence spectrometry (XRF) [6], is among the highly mature spectral and optical techniques for ppm- and ppb-level detection of trace elements in complicated matrices. However, long incubation time, bulky and costly equipment, multi-step sample preparation processes, and requiring chromatographic methods for metal ion speciation are some bottlenecks for real-time monitoring of metal ions using spectral techniques [7]. Thus, electrochemical methods are preferred for determination of trace elements as low as ppm and ppb levels both in situ [8,9] and in vivo [10,11].

The functionality of electrochemical sensors is based on resonant quantum tunnelling, which results in the variation of the resistance of the film while exposed to the analyte [12]. Containing a sensing element and a pair of electrodes, the electrons pass through the electrodes, and the reaction between the sensing layer and the analyte of interest increases the signal amplitude, which can be used for detecting the elements. In recent years, pencil graphite electrodes (PGE) have been used as electrode material in many sensing studies due to their low cost, high omnipresence, disposability, high mechanical stability, and analytical reproducibility (e.g., [13]). PGE is able to provide reproducible, voltametric peaks and a fast rate of electron transfer for usual Fe(CN)_6_^3−/4−^ and Ru(NH_3_)_6_^3+/2+^ redox systems [14]. Despite its other electrochemical benefits (high reactivity, low background current, etc.), PGE is not commonly investigated for trace elements’ detection [7].

Disposition of the sensing layer on the surface of the electrode is another important factor limiting the use of certain materials. For instance, one common issue for electrodeposition of metals on the surface of the electrode, especially those with low reduction potential, is the difficulty of the procedure because of the fractious evolution of hydrogen during electrodeposition [15]. Conductive polymers (CP) are a promising choice, as they mix the properties of metals (e.g., tunable electrical charge transfer) and regular polymers (e.g., the ease of synthesis and high strength) [16,17,18]. The structure of CPs includes a conjugated backbone with alternated single and double comprise π-bonds in which unpaired electrons are able to move freely [19]. Due to the extended π-conjugated backbone structure in CPs as well as the ability to form stable polarons, they are considered as great candidates for supercapacitors [20,21], energy storage [22], and as the sensing layer [23], particularly for metal ion detection [24].

The present work intended to develop a new way for selective determination of Cu (II), Cr (III), and Fe (III) by taking advantage of polyaniline (Pani)-modified PGE in conjunction with PDEIS. To the best of authors’ knowledge, this is the first study that evaluated the potential of PDEIS for metallic ion determination using conductive polymers. In this work, rapid preparation of PAni/PGE was obtained via electropolymerization of aniline on the surface of PGE using cyclic voltammetry (CV). PDEIS was then performed to study the relationship between time, frequency, and potential of the sensing layer in simultaneous alternative current (AC) and direct current (DC) responses. In addition, the decomposition of AC responses into the different potential-dependent elements of the equivalent circuit was scrutinized. After characterization, the performance of the sensing platforms was studied by EIS technique as well. The responses were then correlated with the modified Randles equivalent circuits to assess the selectivity of the sensing layer. As the electrochemical over-oxidization occurs at CPs, to minimize metal-chelate binding, the Nyquist plot was also investigated at the corresponding oxidative potentials.

## 2. Materials and Methods

### 2.1. Materials

Distilled and unmodified aniline (≥99.5%) (sigma chemicals, MO, USA) was purified until it was eventuated to colorless liquids and kept in the dark at 5 °C. Fresh phosphoric acid (sigma chemicals, USA), analytical grade, was used. Oxygen-free nitrogen (OFN) was prepared from Nissan-IOI, Malaysia. All aqueous solutions were newly prepared using ultra-pure water from Milli-Q plus (Millipore Corp., MA, USA). All other chemicals including calcium chloride, potassium chloride, potassium ferricyanide, copper chloride, sodium perchlorate, and chromium nitrate (Merck KGaA, Darmstadt, Germany) were analytical grade and used without any purification or further treatment.

### 2.2. Electrode Fabrication

**Figure 1:**
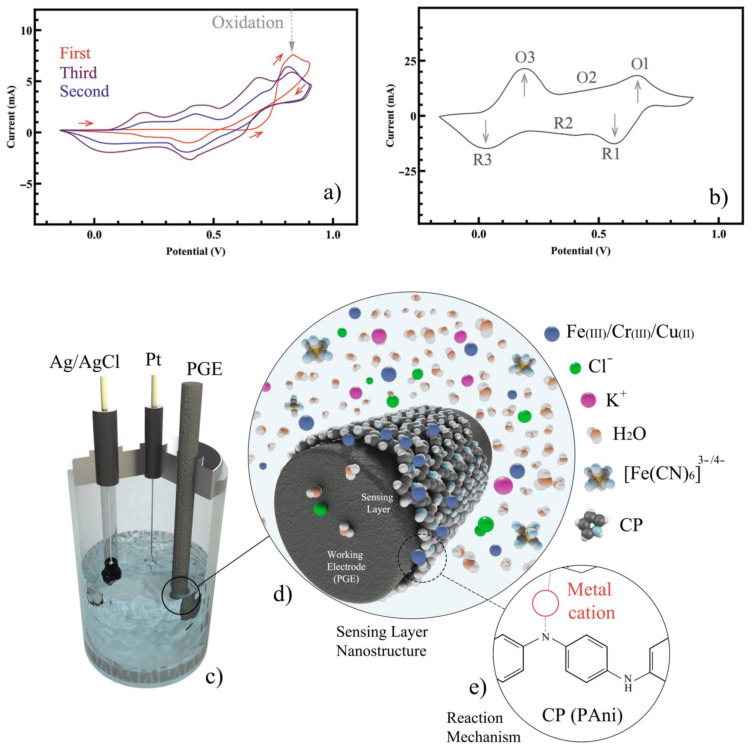
CV of PAni for (**a**) deposition and (**b**) the characterization. (**c**) The schematic of the test setup with the three-electrode cell. (**d**) Deposition of the CP on the surface of the PGE. (**e**) Reaction between PAni and the analyte.

All the electrochemical measurements were conducted by personally controlled and computer-equipped CompactStat potentiostat (Ivium Technologies, Eindhoven, The Netherlands) interfaced with the IviumSoft software package 1.9 for data collection and characterization of CV, EIS, and PDEIS measurements, respectively. The potentiostat plays as the actuator in PDEIS probing. All the reagents were purchased from Sigma Aldrich, ON, Canada, unless it is mentioned. A custom-made, three-neck electrochemical cell was designed and used: The working electrode was the composite 2B pencil graphite (1.8 mm diameter ∅ and 100 mm length, Staedtler Lumograph, Germany), which was covered with aniline monomer. The counter electrode was an external platinum (Pt) wire (Metrohm, ON, Canada), cleaned with a 1:1 H_2_SO_4_/HCl solution. To complete the circuit (to which all potentials are referred), pseudo Ag/AgCl wire was used as the reference electrode. The pseudo Ag/AgCl electrode was synthesized by putting a silver wire with a platinum electrode in a saturated KCl solution and applying the DC voltage of 1.6–1.8 V for 30 min. To remove the impurities and defects, the graphite leads were treated with 5% HNO_3_ overnight, washed with DI water, and dehydrated at room temperature before sealing into the PVC tube. After preparing all the standard electrodes, the electrochemical syntheses and electrodeposition of PAni on 2B pencil graphite (as an unmodified electrode) were potentiodynamically performed by CV on 10 mL aqueous solution, comprised of 0.5 M phosphoric acid (H_3_PO_4_) and 100 mM of aniline at a scan rate of 100 mV/s over applied and sweeping potential ranges (vs. Ag/AgCl) from −0.2 V to + 1.0 V (Figure 1a). This resulted in a greater robustness (in compared to drop-casting) due to the strong bonding between the PAni and PGE. The film thickness was controlled by the number of cycles in CV, as a thick sensing layer resulted in a lower response due to the generation of an additional resistance into the cell.

### 2.3. Experimental Setup, Electrical Measurements, and Optimization

PDEIS was performed within the frequency range of 15 to 1000 Hz by real-time analysis of 20 wavelets at each 2-mV step of a staircase potential ramp with a ramp rate of 2 mV/s (narrower than the probing amplitude) to provide a quasi-continuous scan. In PDEIS, due to the nonstationary characteristics of the system, a low range of frequency was applied, and the potential variation was lower than that of EIS. All the stages were established by superposition of the responses at various phase shifts through the probing stream. EIS assessments of the modified electrode versus the reference electrode were then conducted in another 10 mL solution (consisting of 10 μM of the trace element) by applying a sinusoidal wave of 100 mV in a frequency range of 100 KHz to 0.01 Hz with 50 step/dec under OFN atmosphere at the temperature of 25 ± 2.5 °C. In all experiments, PAni-functionalized PGE was immersed in the stirred solution (300 rpm) containing the metal ion to be accumulated on the electrode surface, and pH values were also taken into account. The EIS was measured 5 min after the sensor was exposed to the analyte of interest so that the ions could freely diffuse through the surface of the electrode. The recording time for each round of experiments was roughly 2 min. The electrode was then removed from the sample solution, rinsed with ultrapure water, and put in the supporting electrolyte. To monitor the electron transfer along the electrode and electrolyte, anionic-probe ion pairs, i.e., $\mathrm{Fe}{\left(\mathrm{CN}\right)}_{6}^{3-}$, was used as the redox probe. Additionally, all experiments were conducted at a constant pH of 3 (as suggested in [25]). Of note, at higher pH values (>6.0), hydrolysis of the ions (due to the formation of hydroxylated species) may interfere with the accumulation of ions at the surface of the electrode. Therefore, the majority of the studies were for the detection of metal cations in acidic environments (e.g., [25,26]). Although the sensors were both reusable after each experiment, fresh films were used for each analyte of interest for a more accurate investigation of the electropolymerized polymers.

### 2.4. Theoretical Background

The standard technique of EIS involves reading the system’s response to a sinusoidal perturbation applied in the form of either voltage $(V\left(t\right)=V_{0}e^{-iwt-\varphi_{v}}$) or current ($I\left(t\right)=I_{0}e^{-iwt-\varphi_{i}}$) (where *ω* is the angular frequency, *V_0_* and *I_0_* are the magnitudes of the oscillating signals, and *φ* is the phase of the complex voltage and current). Following Ohm’s law, impedance (*Z*) is simply calculated as:

(1) $$Z=\frac{V\left(t\right)}{I\left(t\right)}=\frac{V_{0}}{I_{0}}e^{i\left(\varphi_{v}-\varphi_{i}\right)}=Z_{0}e^{i\theta }=Z\;\prime +i\;Z\;\prime \prime$$

**Scheme 1:**
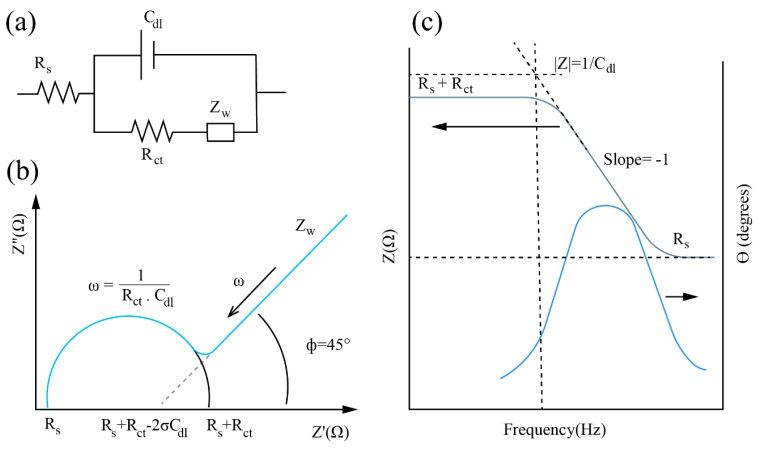
(**a**) The Randles equivalent circuit. (**b**) Typical Nyquist diagram for a faradaic probe. (**c**) The corresponding Bode plot.

Here, the phase of the impedance can be considered as the phase shift (*θ*), which lags the voltage by the applied current, and *Z′* and *Z″* are the real and imaginary parts of the impedance, respectively. In order to interpret the data collected from EIS, an equivalent circuit was commonly utilized to predict the physical, chemical, and biological phenomena occurring in the system. For the faradaic probe (in the presence of a redox pair), the most well-known equivalent circuit is called Randles equivalent circuit (presented in Scheme 1a). It contains the resistance of the solution ($R_{s}$) related to the bulk properties of the electrolyte, the Warburg impedance ($Z_{W}$) for the diffusion of redox, the double-layer capacitance ($C_{dl}$), and the charge-transfer resistance ($R_{ct}$). The last two are related to the surface properties of the interface between the electrode and the solution. The Nyquist and Bode plots are two usual graphical representatives of EIS. In the Bode plot (presenting the impedance and phase angle as a function of frequency), the magnitude of impedance |*Z*| approaches $R_{s}$ and $R_{ct}$ values at very high and very low ranges of the frequency, respectively. The corresponding Nyquist plot (showing the real and imaginary parts of impedance), for a typical Randles circuit is shown in Scheme 1b,c. The symbol σ is the Warburg coefficient, which is affected by the properties of the redox couple and the area of the electrode. In the faradaic impedance, charge transfer is usually considered as the most important factor for electron transfer from the electrode to the electrolyte [27].

The combination of EIS and CV presents potentiodynamic EIS (PDEIS), which correlates the AC and DC perturbation by the analysis of the typical AC responses (the impedance and double layer capacitance) as a function of the potential. The general form of perturbation of the electrode potential in PDEIS is represented as [28]:

(2) $$\Delta i=\left(\frac{\partial I}{\partial V}\right)\Delta V+\left(\frac{\partial I}{\partial C_{s}}\right)\Delta C_{s}+\left(\frac{\partial I}{\partial \alpha }\right)\Delta \alpha$$

where $C_{s}\;$and *α* are the surface concentration of the metal cation and surface coverage of metal adatoms generated by the deposition. While the first term is usually modelled by charge transfer resistance, $R_{ct}\;$(as it corresponds to the active impedance component), the second term is in direct correlation with diffusion (similar to the Warburg impedance) due to the variation of local concentration at different frequencies. Finally, the last term is highly dependent on the reversibility of the electrochemical system [29].

The CV of PAni/PGE along with the schematic of the experimental setup is shown in Figure 1. It is widely accepted that in an acidic environment, aniline radical cations (which were produced at the beginning of oxidation) are paired together in the polymerization process [30]. The cation itself is acidic, so it is expected to remain stable in an acidic environment. The structure of the primary dimers of aniline during oxidation can be head to head, tail to tail, and head to tail (depending on the molar ratio of the species and the pH of the environment) [31]. Polyaniline is the result of the sequential bonding of the aniline monomer units to the polymer chain. The presence of nitrogen atoms in phenyl rings causes different redox forms in the physical properties of polyaniline to occur. Typically, there are three distinct forms depending on the degree of nitrogen oxidation. These forms include a completely reduced form, semi-oxidized form, and fully oxidized form, respectively. As shown in the cyclic voltammogram of aniline (Figure 1a), the anodic peak occurred in the first forward scroll due to the monomer radical cation formation. However, the cathode peak was not displayed in the returned scroll because of the involvement of radical cations together [32]. This indicated the irreversibility of the electropolymerization process. The formation of PAni/PGE was supported by a CV test. For surface characterization of the PAni/PGE and to confirm whether the polyaniline was appropriately synthesized on the graphite, a typical CV was performed by sweeping the voltage from –0.2 to 1.0 V (Figure 1b). The first pair peak (O_3_/R_3_) was observed within 0.0–0.2 V due to the conversion of leucoemeraldine to emeraldine salt. The second pair peak (O_1_/R_1_) at 0.6–0.7 V was attributed to the oxidation of emeraldine salt and (per)nigraniline and, finally, O_1_/R_1_ was the synthesis of quinone. This observation was supported previously for the successful deposition of PAni on PGE as the same values of potential for the peaks were observed at 0.2 V and 0.7 V [33].

## 3. Results and Discussion

### 3.1. Electrochemical Characterization of PAni Modified PGE

**Figure 2:**
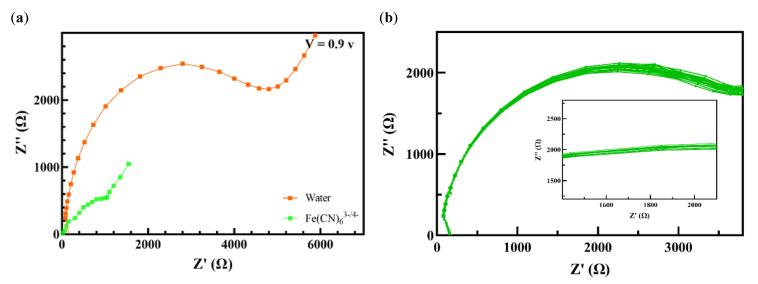
(**a**) The Nyquist spectra of electro-synthesized PAni in the electrolyte and in water for Fe (III) at V = 0.9 v. (**b**) The relative change in the impedance of the sensor after 25 consecutive repetitions.

The role of the electrolyte was initially analyzed by comparing the EIS spectra of one of the selected ions (Fe (III)) in water and in the presence of water at V = 0.9 V (Figure 2a). Not surprisingly, due to the presence of free ions in $\mathrm{Fe}{\left(\mathrm{CN}\right)}_{6}^{3-/4-}$ solutions, the values of impedance at all frequencies were lower than those present in water. Therefore, all the other tests were conducted in the electrolyte. In addition, to show the robustness and stability of the sensing layer, 25 consecutive EIS were conducted under the same conditions, as shown in Figure 2b. The PAni/PGE sensor demonstrated a similar impedimetric response with the standard deviation and relative standard deviation of 83.9925 and 0.012, respectively, for the frequency of 1000 Hz.

**Figure 3:**
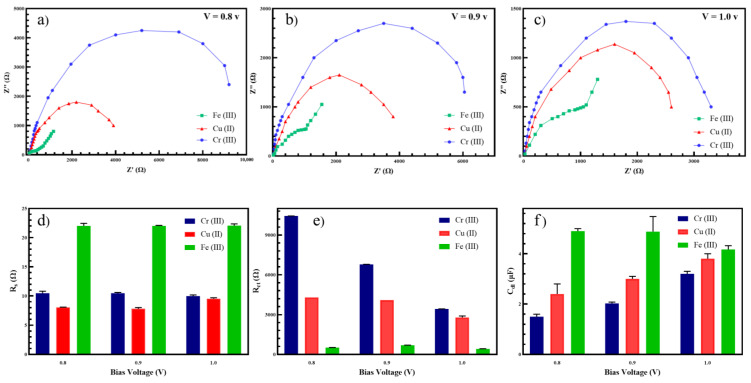
The Nyquist spectra of electro-synthesized PAni in the electrolyte at (**a**) 0.8 V, (**b**) 0.9 V, and (**c**) 1.0 V. The values of (**d**) resistance of solution, (**e**) charge transfer resistance, and (**f**) double layer capacitance for the corresponding equivalent circuit.

Figure 3a–c shows the EIS Nyquist plots of electro-synthesized PAni in the 10 μM Cr (III), Cu (II), and Fe (III) in aqueous media at three different voltages to analyze the effect of voltage on the EIS spectra. The three bias potentials were chosen as 0.8, 0.9, and 1.0 V so it can be within the range of the conducted CV. The results showed the high dependency of the Nyquist plot (impedance values) on the bias voltage, suggesting the necessity to choose the working potential with maximum differentiation between the results. The Nyquist plot of faradaic solutions formed the classic pattern of the depressed semicircle at high frequencies with a straight line as a tail of the circle at low frequencies [34]. Thus, the small diameter of the depressed half circle contributed to the low resistance of the film against the flow of the electrons. The Randles equivalent circuit was considered and its values are presented in Figure 3d–f for $R_{s}$, $R_{ct}$, and $C_{dl}$, respectively. It is obvious that the low value of $R_{ct}\;$represented high charge transfer and redox transformation for the conducting polymer. Additionally, when the Warburg impedance decreased, diffusion of protons and permeability of ions on the surface of the polymer-electrolyte increased, resulting in a better charge transfer. The change in the charge transfer resistance (as a result of changing the voltage) can be explained by [35]:

(3) $$R_{ct}={\left(\frac{\partial i}{\partial V}\right)}^{-1}$$

In the above equation, the potential is rapidly changed to keep the perturbation at the surface constant. Thus, the values of the charge transfer resistance, i.e., the radius of the half circle, were significantly different at different voltages as well as different analytes. As a result, the charge transfer can be used as a promising parameter for feature extraction to selectively differentiate different analytes. Unlike charge transfer resistance, which is in direct correlation with bias voltage, a non-linear regression equation was modelled for the double layer capacitive behaviour of electrochemical studies for a magnitude *K* of current change, as follows [36]:

(4) $$V\left(t\right)-V\left(0\right)=-R_{ct}K\left(1-e^{-t/R_{ct}C_{dl}}\right)$$

Therefore, the value of $C_{dl}$ is not directly related to the bias voltage but generally increases by the increment of the voltages from 0.8 V to 1.0 V (see Figure 3f). Of note, while at low voltages, no degradation or high efficiency was mostly reported and parallel/series issues occurred at high voltages due to the unmatched cell potentials [37]. Therefore, the parallel regions of charges that occurred between the vertical electrodes were disturbed and affected the charge transfer at the liquid–solid interface (double layer) [38]. The different values of $C_{dl}\;$were attributed to the higher interface between iron and the electrolyte than that of copper and chromium.

### 3.2. Underpotential Effect of PAni Modified PGE

**Figure 4:**
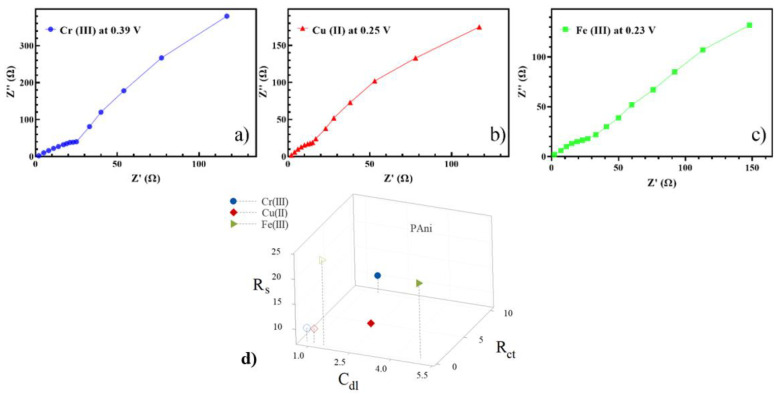
Nyquist spectra of electro-synthesized PAni for the analytes of (**a**) Cr (III), (**b**) Cu (II) and (**c**) Fe (III) at their corresponding reduction potential. (**d**) The values of the elements of the Randles equivalent circuit (filled represents the potential and outline is underpotential).

To study the underpotential effect for PDEIS analysis, the reduction and deposition potentials of the metal ions in the electrodes were also investigated. The reduction potentials of the 10 μM Fe (III), Cr (III), and Cu (II) in an aqueous medium on the modified electrode containing PAni were obtained as 0.39 V, 0.25 V, and 0.23 V for each trace element, respectively. The impedance spectra of the solutions on their corresponding reduction potentials are scrutinized in Figure 4a–c. The charge transfer resistance of Fe (III) containing the solution on its reduction potential was lower than that of Cr (III) and Cu (II) due to the necessity of unpaired electrons in Fe orbitals. In addition, more π-back-donation as a result of a higher electron-rich Fe (III) center resulted in a higher electron transfer of iron ions than those of Cu (II) and Cr (III). Cr (III) was observed to have the largest $R_{ct}\;$due to its larger average particle size, leading to a longer diffusion pathway for electrons, which hindered the electron transfer between the electrolyte and electrode [39].

There was an interaction between the NH group of PAni and Cu (II) that resulted in oxidization of imine group of PAni and the reduction of Cu (II), followed by oxidization through imine groups. Therefore, PAni hosted Cu (II) by replacing a hydrogen bond to PAni-Cu(II) (Figure 1e) [40]. The oxidation of the amine group was also observed by Fe (III) and Cr (III), but, particularly in the presence of $K_{3}[Fe{\left(CN\right)}_{6}]/K_{4}[Fe{\left(CN\right)}_{6}]$, PAni/PGE facilitated the redox reaction of Fe (III) as iron ions were dominant in the solution.

One possible way to show the selectivity of electrochemical sensors with EIS is using the values’ equivalent circuit parameters as the feature extraction. This novel technique was widely described in our recent previous study [41]. In this regard, the values of three main parameters in the Randles equivalent circuit ($R_{ct}$, $R_{s}$, and $C_{dl}$) for the three analytes of interest at the corresponding voltage are represented in Figure 4d. Although this technique can differentiate between the analytes of interest at their potentials, overlapping such features at a broad range of concentrations as well as adjacency of the features led us to represent another technique (i.e., PDEIS) to show the selectivity of the synthesized sensors.

### 3.3. Potentiodynamic Profiles

**Figure 5:**
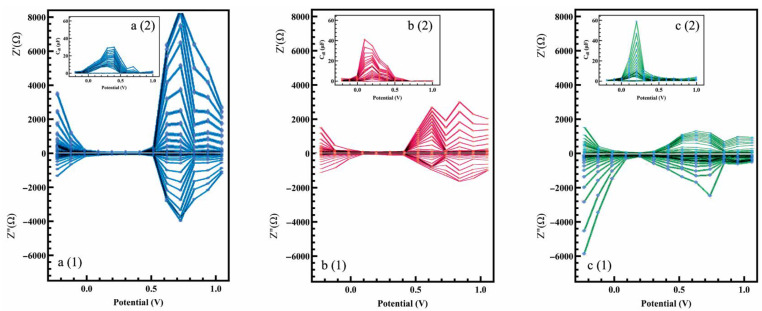
PDEIS spectra (1) impedance and (2) capacitance of electro-synthesized PAni within the potentials of –0.2 V to 1.0 V for (**a**) Cr (III), (**b**) Cu (II) and (**c**) Fe (III).

In electrochemical measurements, a bias signal is initially assigned affecting all the parameters. Therefore, the resulting EIS spectra are snapshots of a stationary system at fixed and constant potential states. However, optimization of the bias potential prior to all the measurements is required to obtain a highly selective and sensitive sensing layer with further efficiency. In this regard, PDEIS was performed to study the change in the system for a range of potentials in the time domain. PDEIS superimposed AC probing at different frequencies (100 kHz to 0.01 Hz) on the staircase potential scan, enabling individual monitoring of interrelated processes on dynamic real-time interfaces. This dynamic mode was based on frequency response acquisition of the variation in equivalent circuit parameters (particularly double layer capacitance) [28]. Figure 5 shows the PDEIS spectra of Cr (III), Cu (II), and Fe (III) on PAni-modified PGE, respectively, at the sweeping dynamic voltages from 1.0 to –0.2 V. As the impedance signals at adjacent potentials were smoothly connected, no abrupt steps were seen in the waveform. Each probe in the potential axis connected the points of equal frequency associated with the Nyquist plot. The AC components (impedance and capacitance) were decomposed and showed potential dependences. The magnitude of the impedance and other electrochemical parameters was the function of the applied potential of the conductive polymer. It should be noted that another common representation of PDEIS is in a 3-D plot, including the real and imaginary values of impedance and potential at three axes, as it was presented in [29,42].

The first observation on Figure 5 is the significant difference on the spectra of Cu (II), Fe (III), and Cr (III). It was reported that PAni is highly resistive at negative and sufficiently positive potentials [43]. The solution of Cr (III) showed the least resistivity (impedance) while the potential was within the range of V = 0.1 V to 0.4 V (see Figure 5a(1)). These values showed the optimum potential in which PAni/PGE showed the least impedimetric behaviour, suggesting the higher charge transfer between the chromium ions and the sensing layer. Then, the real and imaginary parts of the impedance rose dramatically while reaching to the highest values at V = 0.7 V. There was no linear relationship between the magnitude of the impedance and capacitance, meaning that the lowest value of the capacitance did not necessarily take place at the same potential where the highest magnitude of the real and imaginary parts of the impedance occurred (see Figure 5a(2)). This provided two significant parameters (absolute value of impedance and $C_{dl}$) to be used as features to separate different analytes in a mixture at high and at low potentials, respectively.

As shown in Figure 5b(1), the PAni film also showed a smaller range of low impedance when it was exposed to the Cu (II) solution (as compared to Cr (III)). The same range of potentials (V = 0.1 V to 0.4 V) with the lowest resistivity was obtained for Cu (II). Higher stored electric energy was at the interface of the Cu (II) electrode, and the electrolyte was also observed between V = 0.3 V to 0.6 V (Figure 5b(2)). The PAni film exposed to Fe (III) showed a very high impedance value at negative potentials, which slowly decreased until it reached its lowest value at V = 0.2 V (Figure 5c(1)). The sharp peak observed at the double layer capacitance profile of Fe (III) was attributed to the generation and subsequent oxidation of Fe nuclei (Figure 5c(2)). Similar to conventional cyclic voltammetry, it was crystal clear that the potentiodynamic profile of the modified electrodes exposed to the trace elements was totally distinct. As PDIES provides the synergetic characteristics of CV and EIS, it provides a distinguished selectivity to the electrochemical sensors in comparison to standalone EIS represented in the previous section.

PDEIS provides two major advantages over other common methods for metal ion determination: (1) faster response in comparison to optical methods as, for example, ultraviolet–visible spectroscopy and fluorescence studies have long response times from 20 min to 2–3 h [44] and (2) among electrochemical methods with comparable limit of detection (LOD), the recent studies require surface modification by adding a dopant or heterojunction structure while this method can achieve a similar LOD without any impurities. e.g., [45,46]. While all the experiments were conducted at the concentration of 10 µM to prove the applicability of PDEIS, we expected that the sensing layer modification could result in improving the detection mechanism in a broad linear range with high sensitivity. In our next step, we are trying to use nanocomposites (the heterojunction between PAni and carbonaceous materials and/or metal oxides) and use the same technique for a broad range of concentrations in the presence of multiple ions.

## 4. Conclusions

In this paper, PDEIS was used to synthesize an inexpensive, disposable, and renewable electrochemical chemiresistor (fabricated using the pencil tip), selective toward three common trace metals (i.e., Cr (III), Cu (II), and Fe (III)). EIS was also used to characterize and study the conductivity and charge transfer resistance of the thin film of PAni in the aqueous medium containing these metals. The repulsion between the cationic metal ions and polymer was balanced by the chelating dopant, which generated the escalated background currents due to the polymer redox and charging reactions. At various applied potentials, Fe (III) revealed the highest charge transfer and double layer capacitance due to its orbital configuration and paramagnetic property, both of which were selected as the feature extraction for further selectivity. PDEIS was then introduced for improving the selectivity of the chemiresistors by differentiating between the metal ions with very low interference. This study showed that PDEIS can provide unique profiles for different trace elements by combining the characteristics of CV and EIS. Future work on this technique will be aimed at further characterization of the sensing layer (by imaging techniques) and elucidating the application of PDEIS in real clinical samples. In addition, the potential of PDEIS on the detection of metal ions in the mixture at a broad range of concentrations will also be tested.
